# A new druggable target for clinical management of COPD

**Published:** 2013-11

Exposure to tobacco smoke and environmental pollutants can cause chronic obstructive pulmonary disease (COPD), a severe lung disease characterised by progressive breakdown of lung tissue (emphysema). COPD is a leading cause of death worldwide and is described as a global epidemic. SESN2, an antioxidant protein, has been implicated in genetically determined emphysema. To find out if this protein is also involved in COPD pathogenesis, Harald von Melchner and colleagues knocked out *Sesn2* in a mouse model of cigarette-smoke-induced pulmonary emphysema. They discovered that inactivation of *Sesn2* protects mice from the disease. Furthermore, they demonstrate that protection is mediated by upregulation of PDGFRβ signalling, which has a role in the maintenance of alveoli. The importance of SESN2 in disease pathogenesis is supported by the group’s finding that SESN2 is overexpressed in the lungs of humans with advanced COPD. These results implicate SESN2 as a key biomarker and potential therapeutic target in the clinical management of COPD. **Page 1378**

